# ﻿Two new species of the genus *Joeropsis* Koehler, 1885 (Isopoda, Asellota, Joeropsididae) from Korean waters

**DOI:** 10.3897/zookeys.1090.80149

**Published:** 2022-03-24

**Authors:** Sung Hoon Kim, Jong Guk Kim, Seong Myeong Yoon

**Affiliations:** 1 Division of Ocean Sciences, Korea Polar Research Institute, Incheon 21990, Republic of Korea; 2 Marine Ecosystem Research Center, Korea Institute of Ocean Science and Technology, Busan 49111, Republic of Korea; 3 Department of Biology, College of Natural Sciences, Chosun University, Gwangju 61452, Republic of Korea; 4 Educational Research Group for Age-associated Disorder Control Technology, Graduate School, Chosun University, Gwangju 61452, Republic of Korea

**Keywords:** Isopoda, *Joeropsis*, morphology, new species, South Korea, taxonomy

## Abstract

Two new species, *Joeropsisdenticulatus***sp. nov.** and *Joeropsissemicircularis***sp. nov.** are reported from South Korea. *Joeropsisdenticulatus***sp. nov.** can be identified by the following character states: whole body has dark brown chromatophores dorsally, lateral margins of the cephalon are smooth and narrowing anteriorly, and basis of pereopods has serrated cuticular scales superiorly. *Joeropsissemicircularis***sp. nov.** can be identified by the following character states: the cephalon, pereonite 4, and pleotelson have dark brown chromatophores dorsally, lateral margins of the cephalon are smooth and parallel each other, and flagellar article 1 of the antenna is swollen and semi-circular in shape.

## ﻿Introduction

The genus *Joeropsis* Koehler, 1885 including 77 species is the largest genus in the family Joeropsididae Nordenstam, 1933 (Boyko et al. 2008). *Joeropsis* can be distinguished from other joeropsidid genera by having a laterally parallel body shape, a dorsally smooth or finely granular body surface, and a not medially expanded maxillipedal palp article 3 (Just 2001; Bruce 2015). Although, occasionally, the colour pattern fades of a body for old preserved specimens or shows intraspecific variations in some species, the pattern of the body has been recognized as an apparent character in *Joeropsis* taxonomy to distinguish species (Menzies 1951; Bruce 2015). Additionally, Bruce (2015) has recently mentioned that shapes of the cephalon, pseudorostrum, maxilliped, pleotelson, male pleopod 1, and uropods can be useful diagnostic characters in this genus.

The genus *Joeropsis* is common in shallow coastal waters, particularly, colonizing in coral reef habitats (Bruce 2009, 2015). The genus is known to be distributed worldwide and is well represented in tropical regions such as the Indo-West Pacific (Kensley and Schotte 2002; Bruce 2009, 2015). Although the genus represents the highest species diversity in the Pacific Ocean with 33 recorded species (Boyko et al. 2008), only nine species have been reported from the North Pacific (Miller 1941; Menzies 1951; Schultz 1966; Kensley 1989; Kussakin 1999; Nunomura 1999). In particular, our knowledge on the genus is relatively poor in the temperate Far East, and only four species have been recorded: *J.affinis* Kussakin, 1961 from the middle Kuril Islands, Russia; *J.lata* Kussakin, 1961 from the Western Kamchktka Shelf, Russia; *J.lobota* Richardson, 1899 from Osaka Bay, Japan; and *J.latiantennata* Nunomura, 1999 from the Shikine Island, Japan (Kussakin 1961; Nunomura and Nishimura 1976; Nunomura 1999). During surveys of Korean isopods, the authors found two apparently undescribed *Joeropsis* species from sublittoral habitats. Here, we provide detailed descriptions and illustrations of these two species.

## ﻿Material and methods

**Figure 1.**
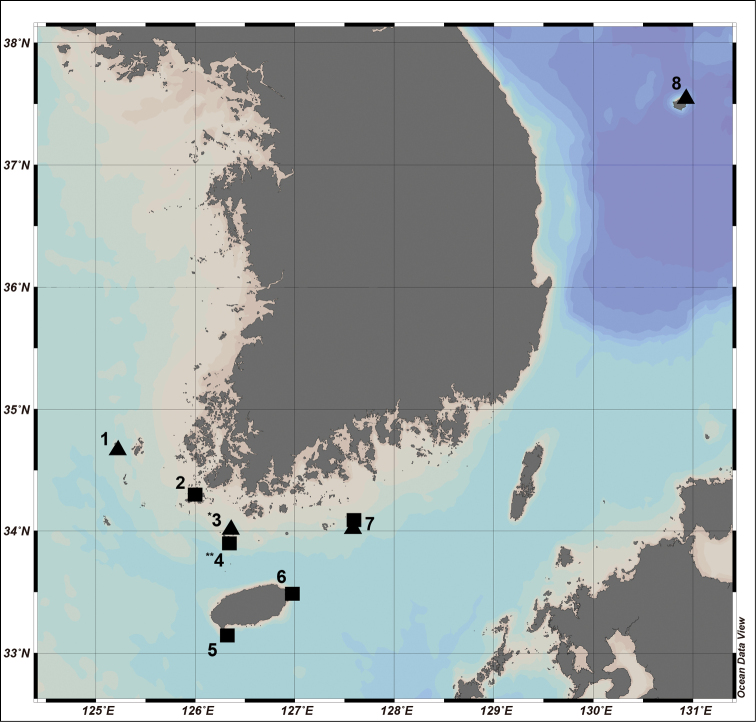
Map of the sampling stations of *J.denticulatus* sp. nov. (filled square, ■) and *J.semicircularis* sp. nov. (filled triangle, ▲). Station numbers of sampling localities (also type localities) are listed in Table 1.

Materials of *J.denticulatus* sp. nov. and *J.semicircularis* sp. nov. were collected from eight sampling stations of the sublittoral zones in Korean waters using a Smith-McIntyre grab and SCUBA diving (Fig. 1; Table 1). The substrate was gravel mud flat with depths of 10 to 30 m. Collected material was sorted using a sieve with a 1 mm mesh size and immediately fixed with 94% ethyl alcohol. After transferring this material to the laboratory, observation was conducted under a dissecting microscope (Olympus SZH-ILLD) and a compound microscope (Olympus BX50). Measurements and drawings of specimens were carried out with the aid of a drawing tube. Terminology for body and appendage morphology follows Bruce (2009, 2015). Drawings were digitally scanned, inked, and arranged using a tablet and Adobe Illustrator CS6 as described by Coleman (2003, 2009). Examined materials in this study were deposited at the National Institute of Biological Resource (**NIBR**) and Chosun University in South Korea.

**Table 1.** Sampling stations of the two new species in Korean waters.

No.	Locality	Geographical Coordinates	Depth (m)	Collecting method	Date
1	Jeollanam-do, Sinan-gun, Heuksan-myeon, Hondo-ri, Hongdo Island	34°40'09"N, 125°10'59"E	10	SCUBA diving	19 Jun. 2018
2	Jeollanam-do, Sinan-gun, Jangsan-myeon, Baegyado Island	34°22'24"N, 126°00'15"E	10	SCUBA diving	12 Apr. 2018
*3	Jeju-do, Jeju-si, Chuja-myeon, Chujado Island	33°59'08"N, 126°19'08"E	10	SCUBA diving	06 Jul. 2019
**4	Jeju-do, Jeju-si, Chuja-myeon, Chujado Island	33°55'18"N, 126°19'27"E	20	Smith-McIntyre grab	17 Apr. 2019
5	Jeju-do, Seoqwipo-si, Daejeong-eup	33°11'24"N, 126°16'08"E	30	Smith-McIntyre grab	31 Jan. 2018
6	Jeju-do, Jeju-si, Udo-myeon, Udo Island	33°31'38"N, 126°57'14"E	15	Smith-McIntyre grab	17 Apr. 2019
7	Jeollanam-do, Yeosu-si, Samsan-myeon, Sangbaeckdo Island	34°03'15"N, 127°35'00"E	15	SCUBA diving	28 Jun. 2017
8	Gyeongsangbuk-do, Ulleung-gun, Buk-myeon, Cheonbu-ri, Gwaneumdo Islet off Ulleungdo Island	37°32'43"N, 130°55'22"E	20	SCUBA diving	19 Jun. 2016

*, type locality of *Joeropsissemicircularis* sp. nov.; **, type locality of *J.denticulatus* sp. nov.

## ﻿Taxonomy

### ﻿Order Isopoda Latreille, 1817

**Suborder Asellota Latreille, 1802**

**Superfamily Janiroidea G.O. Sars, 1897**

#### Family Joeropsididae Nordenstam, 1933

##### ﻿Genus Joeropsis

Taxon classificationAnimaliaIsopodaJoeropsidae

Koehler, 1885

0EA47612-3FBF-56DB-A9D2-70EBAA47117C

Joeropsis Koehler, 1885: 7; Kensley and Schotte 1989: 87; Wilson 1997: 86; Kussakin 1999: 12; Just 2001: 304; Kensley and Schotte 2002: 1428.
Jæropsis : Richardson 1905: 476; Stebbing 1905: 50.
Jaeropsis : Nordenstam 1933: 191; Menzies and Barnard 1959: 10; Menzies 1962: 64; Menzies and Glynn 1968: 76.
Iaeropsis : Nierstrasz 1941: 288 (unjustified emendation).

###### Type species.

*Joeropsisbrevicornis* Koehler, 1885 by original designation.

###### Diagnosis.

Body smooth, rarely with sculpture dorsally, laterally parallel. Pseudorostrum with overhanging apex. Eyes positioned dorsolaterally. Mandibles with evenly spaced cusps; spine row consisting of long setae. Maxilliped, endite reaching end of palp article 3; palp article 3 lacking medial lobe. Pereopod 1 with 2 claws and pereopods 2–7 with 2 or 3 claws. Pleopod 2 with a few short simple setae distally in females. Pleopod 3, exopod obliquely articulated between articles (Just 2001; Bruce 2015).

###### Remarks.

The genus *Joeropsis* can be differed from other joeropsidid genera by having a dorsally smooth or finely granular body (vs. coarsely granular and nodular in *Rugojoeropsis* Just, 2001) and parallel lateral body shape (vs. converging posteriorly in *Scaphojoeropsis* Just, 2001) (Bruce 2015). The colour pattern of a body can be distinguishable to easily separate members of the genus (Bruce 2015). Additionally, the morphology of the cephalon, pseudorostrum, antennae, maxilliped, pleotelson, and male pleopod 1 can be the most diagnostic characters for *Joeropsis* species (Kensley and Schotte 2002; Bruce 2009, 2015).

##### ﻿ Joeropsis denticulatus sp. nov.

Taxon classificationAnimaliaIsopodaJoeropsidae

6931AAEF-2009-5042-896D-127853049855

http://zoobank.org/0835482B-A1B9-4B6C-A73A-53BC7DAE399A

Figs 2A , 3 , 4 , 5

###### Material examined.

***Holotype*, designated here**: South Korea • 1 ♂ (5.0 mm); Jeju-do, Jeju-si, Chuja-myeon, Chujado Island; 33°55'18"N, 126°19'27"E; 20 m; 17 Apr. 2019; Smith-McIntyre grab; NIBRIV0000862803.

***Paratypes***: 2 ♂♂ (4.2, 4.8 mm), 3 ♀♀ (3.6, 3.8, 3.8 mm); same data as for holotype; NIBRIV0000896084.

###### Additional material.

South Korea • 1 ♂; Jeju-do, Jeju-si, Udo-myeon, Udo Island; 33°31'38"N, 126°57'14"E; 17 Apr. 2019; 15 m; Smith-McIntyre grab • 5 ♂♂, 4 ♀♀; Jeju-do, Seoqwipo-si, Daejeong-eup; 33°11'24"N, 126°16'08"E; 31 Jan. 2018; 30 m; Smith-Mclntyre grab; NIBRIV0000862803 • 1 ♂; Jeollanam-do, Sinan-gun, Jangsan-myeon, Baegyado Island; 34°22'24"N, 126°00'15"E; 12 Apr. 2018; 10 m; SCUBA diving • 1 ♂; Jeollanam-do, Yeosu-si, Samsan-myeon, Sangbaeckdo Island; 34°03'15"N, 127°35'00"E; 15 m; 28 Jun. 2017; SCUBA diving.

###### Etymology.

The specific name, *denticulatus*, is derived from the Latin word *denticulatus*, meaning “with small teeth”. This name refers to pereopods possessing serrate cuticular scales.

###### Description of holotype male.

**Figure 2.**
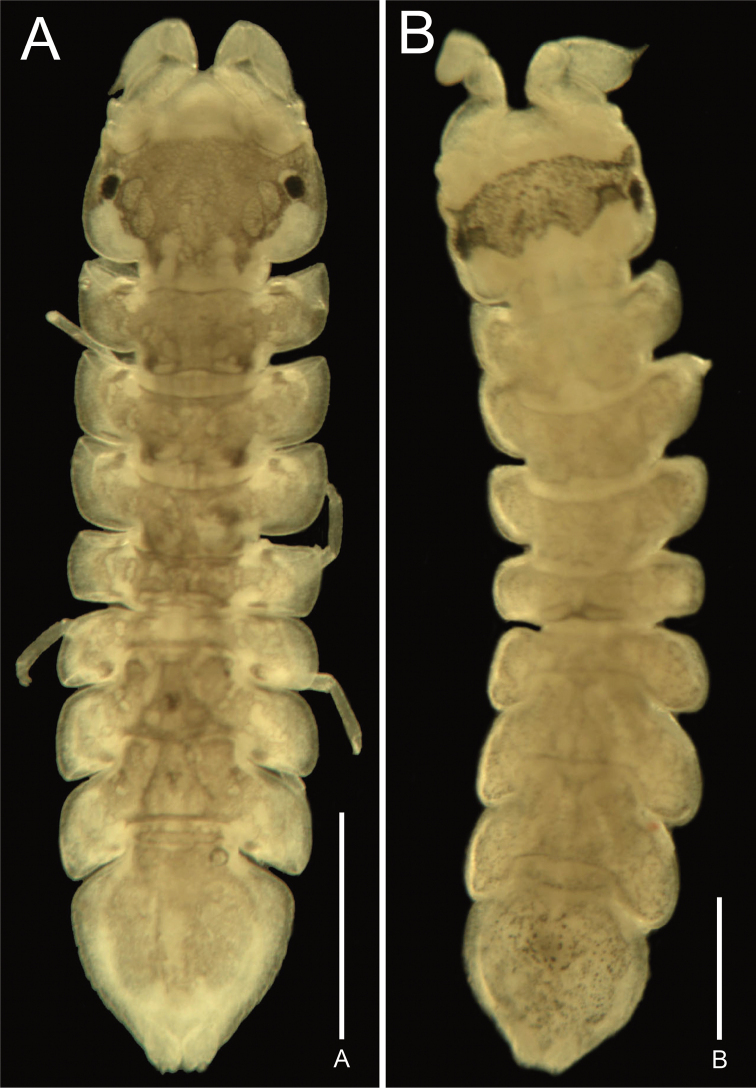
Photographs of two new species of *Joeropsis*: **A***J.denticulatus* sp. nov., one of paratypes (NIBRIV0000896084), dorsal view **B***J.semicircularis* sp. nov., one of paratypes (NIBRIV0000896085), dorsal view. Scale bars: 10 mm (**A**); 5 mm (**B**).

**Figure 3.**
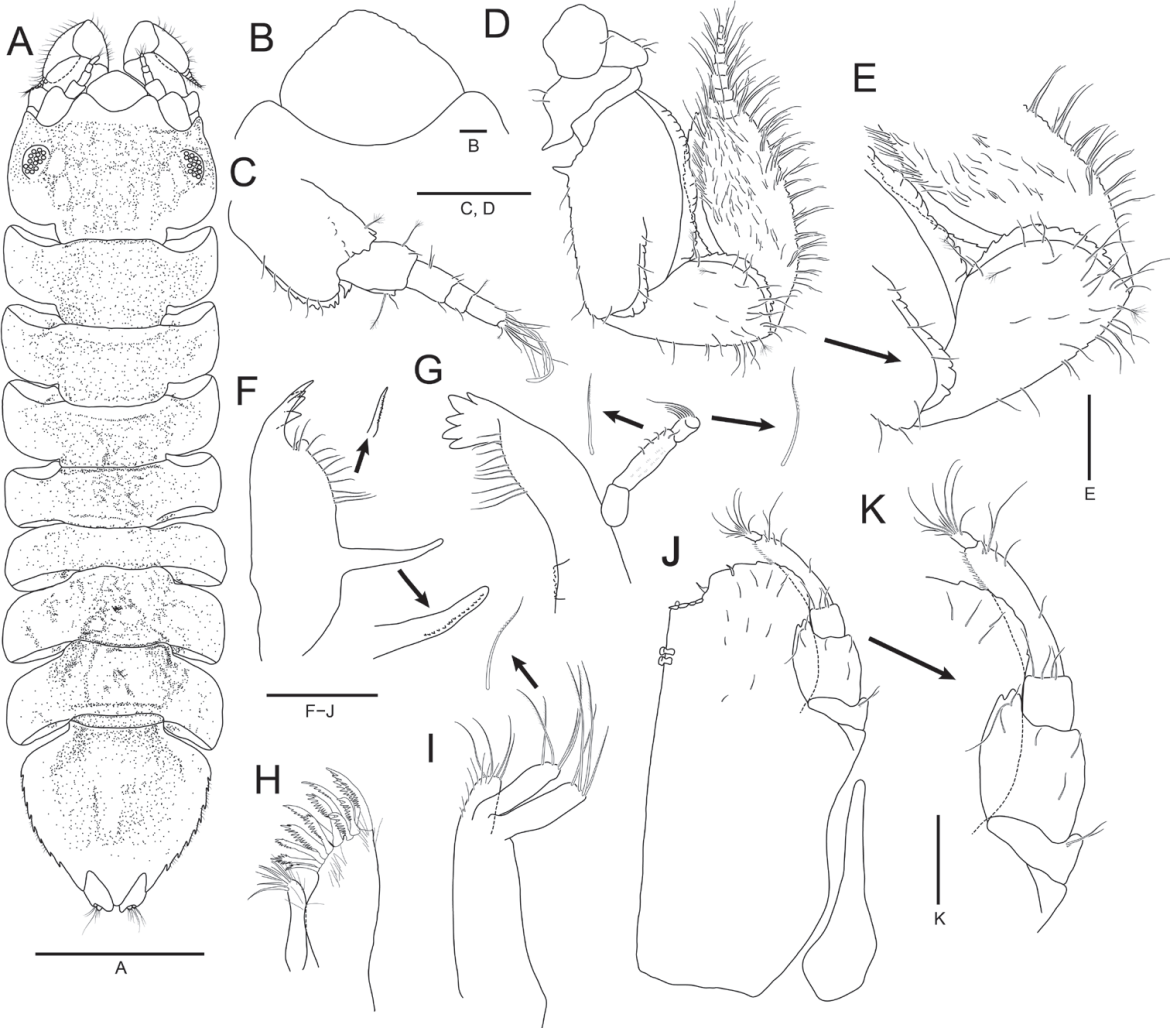
*Joeropsisdenticulatus* sp. nov., holotype, male **A** habitus, dorsal view **B** pseudorostrum **C** antennula **D** antenna **E** detail of peduncular articles 5 to flagellar article 1 **F** left mandible **G** right mandible **H** maxillula **I** maxilla **J** maxilliped **K** detail of maxillipedal palp. Scale bars: 1 mm (**A**); 0.2 mm (**C, D, F–J**); 0.1 mm (**E, K**); 0.05 mm (**B**).

***Body*** (Figs 2A, 3A) almost 3.9 × longer than width; dorsal surface matte, smooth, without setae. ***Cephalon*** 0.7 × as long as wide; lateral margins narrowing anteriorly; eyes positioned sublaterally, globular, dark brown, dorsally bulging. ***Pseudorostrum*** (Fig. 3B) 0.7 × as long as proximal wide, narrowing anteriorly; apex rounded. ***Pereonites*** not compact; lateral margins smooth. ***Pleotelson*** ~ 1.0 × longer than greatest width, shield-shaped, tapering distally; caudomedial lobe subacute, tapering distally; lateral margins slightly convex, with 8 spines.

***Antennula*** (Fig. 3C), peduncular article 1 rectangular, 1.4 × longer than width, with serrate cuticular scales distally, 5 simple setae along with lateral margin, 1 penicillate seta distally, and 5 penicillate setae medially; article 2 oblong, 0.5 × as long as article 1, with 1 simple seta and 2 penicillate setae distally, and cuticular scales laterally; article 3 oblong, 0.6 × as long as article 2, with 2 simple setae laterally and 3 simple setae distally; flagellar article 1 almost 0.5 × as long as peduncular article 3, with 1 penicillate seta and 1 simple seta distally; article 2 ~ 2.0 × longer than flagellar article 1, with 2 simple setae and 2 aesthetascs distally, and 1 simple seta laterally; article 3 minute, with 4 simple setae, 1 penicillate seta, and 1 aesthetasc on distal end. ***Antenna*** (Fig. 3D, E) with 10 flagellar articles; peduncular article 3 with 1 process on medial margin; article 5 1.4 × longer than articles 1–4 combined, with serrate lateral cuticular scales and 1 medial process proximally; article 6 0.7 × as long as article 5, widening distally, with serrate cuticular scales, 3 penicillate setae, and several simple distal setae; flagellum with numerous simple setae; flagellar article 1 elongate ovoid, 1.8 × longer than remaining articles combined, 1.3 × longer than peduncular article 6, with cuticular scales laterally.

***Mandibles*** (Fig. 3F, G), molar process distal half finely serrated; incisor with 5 cusps; palp article 2 with serrate setae distally; palp article 3 with serrate setae along with lateral margin. ***Left mandible*** (Fig. 3F) with a protrusion between incisor and molar process; spine row composed of 12 serrate setae. ***Right mandible*** (Fig. 3G), spine row consisting of 10 serrate setae. ***Maxillula*** (Fig. 3H), mesial lobe with 3 robust simple setae and several fine setae distally; lateral lobe with 12 strongly serrate robust setae and several fine setae on distal region. ***Maxilla*** (Fig. 3I), mesial lobe shorter than other lobes, with 4 simple setae distally and fine setae along with medial margin; mesial and outer lobes with 4 serrate setae distally. ***Maxilliped*** (Fig. 3J, K), endite expanding half of palp article 4, with several short simple setae on medial surface and 2 coupling hooks on medial distal end; distal region of endite rounded and serrated while concave medially, with 4 medial tubercular robust setae; palp article 1 with 2 simple setae distally, article 2 2.8 × longer than article 1, with distally bifid mesial lobe, article 3 square, 0.4 × as long as article 2, with several simple setae distally, article 4 3.0 × longer than article 3, with fine setae on medial margin and several setae laterally, article 5 0.2 × as long as article 4, with several simple setae on distal end; epipod 3.4 × longer than basal width, tapering distally.

**Figure 4.**
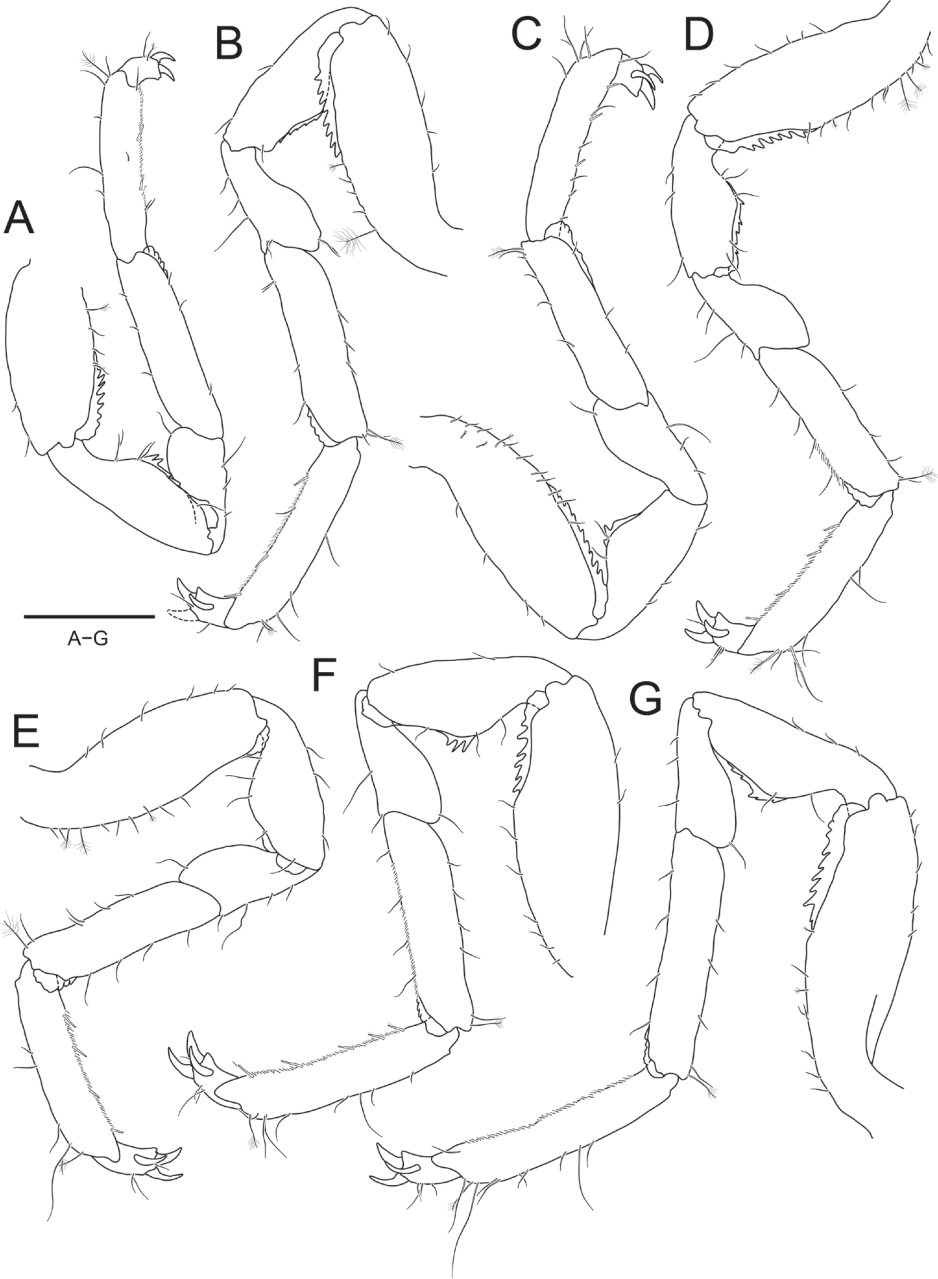
*Joeropsisdenticulatus* sp. nov., holotype, male **A** pereopod 1 **B** pereopod 2 **C** pereopod 3 **D** pereopod 4 **E** pereopod 5 **F** pereopod 6 **G** pereopod 7. Scale bar: 0.2 mm.

***Pereopods*** (Fig. 4A–G), basis and ischium with serrated cuticular scales superodistally; carpus with slightly serrated cuticular scales inferodistally; propodus with 2–4 robust setae inferiorly and numerous short simple setae along with inferior margin. ***Pereopod 1*** (Fig. 4A), basis with 1 penicillate seta superiorly; ischium subequal to basis in length, narrowing proximally; merus 0.6 × as long as ischium; carpus 1.7 × longer than merus; propodus 0.9 × as long as carpus, with 1 penicillate seta on superior distal angle; dactylus 0.2 × as long as propodus, with 2 claws and 1 penicillate seta distally. ***Pereopods 2–7*** (Fig. 4B–G) similar to each other; basis longer than ischium, with 0–2 penicillate setae on superior margin; ischium convex superomedially; carpus ~ 2.0 × longer than merus, with 1 penicillate seta superodistally; propodus similar to carpus in length, with 1 penicillate seta superodistally; dactylus ~ 0.2 × as long as propodus, with 3 claws distally.

**Figure 5.**
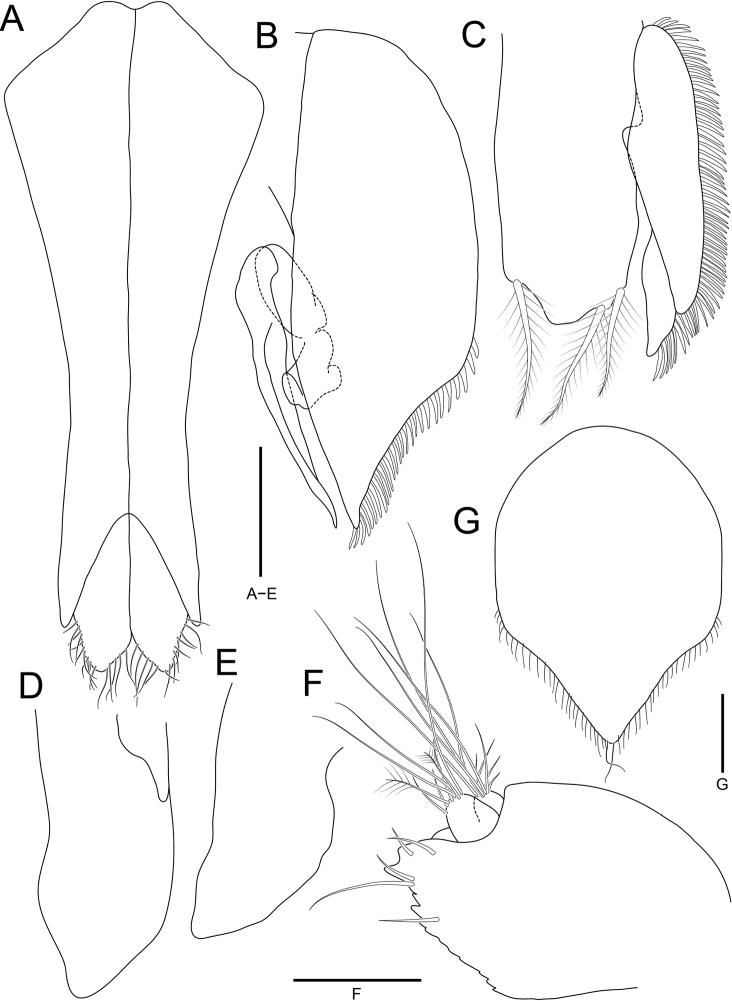
*Joeropsisdenticulatus* sp. nov., holotype, male **A** pleopod 1 **B** pleopod 2 **C** pleopod 3 **D** pleopod 4 **E** pleopod 5 **G** uropod. Paratype, female: **F** pleopod 2. Scale bars: 0.2 mm (**A–E, G**); 0.1 mm (**F**).

***Pleopod 1*** (Fig. 5A) 2.6 × longer than greatest width, slightly concave laterally, narrowing distally; distolateral lobe projected, extending distally; apical lobe rounded, with marginal simple setae distally. ***Pleopod 2*** (Fig. 5B), protopod 2.7 × longer than mid-width, concave distolaterally bearing cuticular scale-setae, with subacute distal end; endopod positioned at 0.7 length of protopod from proximal region; exopod curved outwardly; appendix masculina reaching distal end of protopod, tapering distally. ***Pleopod 3*** (Fig. 5C), endopod 2.1 × longer than width, with 3 plumose setae distally; exopod with cuticular scale-setae along with lateral margin, first article 3.8 × longer than width; second article 0.4 × longer first article. ***Pleopod 4*** (Fig. 5D), endopod 2.2 × as long as wide, tapering distally; exopod vestigial. ***Pleopod 5*** (Fig. 5E) without exopod; endopod 2.0 × as long as wide, tapering distally.

***Uropods*** (Figs 3A, 5F), protopod extending beyond margin of pleotelson, with strongly produced distal end, serrated medial margin, and 5 simple setae on distal region; endopod with 2 penicillate setae and several simple setae distally; exopod smaller than endopod, with several simple setae distally.

###### Description of female.

Female similar to male. ***Pleopod 2*** (Fig. 5G) 1.4 × longer than greatest width, tapering distally, with fine setae on distal region; apex subacute, with 2 simple setae.

###### Size.

Males 4.2–5.0 mm, mean 4.7 mm (*n* = 3); females 3.6–3.8 mm, mean 3.7 mm (*n* = 3); all from type series.

###### Colour pattern.

Whole body is covered with dark brown chromatophores (Figs 2A, 3A).

###### Variation.

The colour pattern of the body varies between individuals, especially on pereonite 5. Dark brown chromatophores are occasionally faint or not observable on it.

###### Distribution.

South Korea (southern coast of the Korean Peninsula).

###### Remarks.

*Joeropsisdenticulatus* sp. nov. can be identified by the following character states: (1) whole body has chromatophores dorsally; (2) lateral margins of the cephalon are smooth and narrowing anteriorly; and (3) basis of pereopods has serrated cuticular scales superiorly.

Among the total of 77 species, *Joeropsisdenticulatus* sp. nov. is similar to five *Joeropsis* species in having the laterally smooth and narrowing cephalon, anteriorly rounded pseudorostrum, medially lobed maxillipedal palp article 2, laterally serrated pleotelson, and medially serrated uropods: *J.adusta* Bruce, 2015; *J.affinis* Kussakin, 1961; *J.brevicornis* Koehler, 1885; *J.dubia* Menzies, 1951; and *J.salvati* Müller, 1989. Among them, *J.denticulatus* sp. nov. most closely resembles *J.dubia* in terms of pereopods with serrated cuticular scales on ischium, but the former can be distinguished from the latter by having pereopod 1 bearing serrated cuticular scales on the basis (vs. lacking serrated cuticular scales in the latter) and distally rounded pleopod 1 in males (vs. distally triangular in the latter) (Menzies 1951; Kussakin 1999). The new species can be distinguished from the remaining four species in terms of maxillipedal palp article 2 (having distally bifid medial lobe vs. having not in the latter species) and pereopods (having serrated cuticular scales on the basis in the former vs. lacking in the latter species) (Koehler 1885; Bocquet and Lemercier 1958; Amar 1961; Kussakin 1961, 1999; Kensley 1975; Müller 1989; Bruce 2015). A detailed comparison of *Joeropsis* species mentioned above is provided in Table 2.

**Table 2.** Comparison of diagnostic characters among *Joeropsis* species.

Species	Cephalon, lateral margins	Pseudorostrum, apex	Antenna, flagellar article 1	Maxilliped, medial lobe in palp article 2	Pleotelson, lateral margins	Uropod, medial margin
*J.denticulatus* sp. nov.	Anteriorly narrowing/ smooth	Rounded	Not swollen	Present	Serrated	Serrated
*J.semicircularis* sp. nov.	Anteriorly narrowing/ smooth	Rounded	Not swollen	Present	Serrated	Serrated
*J.adusta*	Anteriorly narrowing/ smooth	Rounded	Not swollen	Present	Serrated	Serrated
*J.affinis*	Anteriorly narrowing/ smooth	Rounded	Not swollen	Present	Serrated	Serrated
*J.brevicornis*	Anteriorly narrowing/ smooth	Rounded	Not swollen	Present	Serrated	Serrated
*J.dubia*	Anteriorly narrowing/ smooth	Rounded	Not swollen	Present	Serrated	Serrated
*J.salvati*	Anteriorly narrowing/ smooth	Rounded/ concave	Not swollen	Present	Serrated	Serrated
*J.dollfusi*	Parallel/ smooth	Rounded	Not swollen	Absent	Serrated	Serrated
*J.latiantennata*	Parallel/ smooth	Rounded	Swollen	Absent	Serrated	Not serrated
*J.stebbingi*	Parallel/ smooth	Rounded	Not swollen	Absent	Serrated	Serrated
*J.wolffi*	Parallel/ smooth	Rounded	Not swollen	Absent	Serrated	Serrated

##### ﻿ Joeropsis semicircularis sp. nov.

Taxon classificationAnimaliaIsopodaJoeropsidae

9D44C608-2B25-533A-84BE-7FC84539C063

http://zoobank.org/2585142C-F02D-4951-B429-DF2321053C65

Figs 2B , 6 , 7 , 8

###### Material examined.

***Holotype*, designated here**: South Korea • 1 ♂ (3.7 mm); Jeju-do, Jeju-si, Chuja-myeon, Chujado Island; 33°59'08"N, 126°19'08"E; 10 m; 06 Jul. 2019; SCUBA diving; NIBRIV0000862804.

***Paratypes***: 3 ♂♂ (3.7, 4.0, 4.0 mm), 6 ♀♀ (3.3, 3.4, 3.5, 3.5, 3.6, 3.7 mm), same data as holotype; NIBRIV0000896085.

###### Additional material.

South Korea • 1 ♂; Jeollanam-do, Sinan-gun, Heuksan-myeon, Hondo-ri, Hongdo Island; 34°40'09"N, 125°10'59"E; 10 m; 19 Jun. 2018; SCUBA diving • 1 ♂, 1 ♀; Jeollanam-do, Yeosu-si, Samsan-myeon, Sangbaeckdo Island; 34°03'15"N, 127°35'00"E; 15 m; 28 Jun. 2017; SCUBA diving • 1 ♂, 4 ♀♀; Gyeongsangbuk-do, Ulleung-gun, Buk-myeon, Cheonbu-ri, Gwaneumdo Islet off Ulleungdo Island; 37°32'43"N, 130°55'22"E; 20 m; 19 Jun. 2016; SCUBA diving.

###### Etymology.

The specific name, *semicircularis* is derived from the combination of Latin words *semis*, meaning “a half”, and *circularis*, meaning “round”. This name refers to the first flagellar article of the antenna that is semi-circular in shape.

###### Description of holotype male.

**Figure 6.**
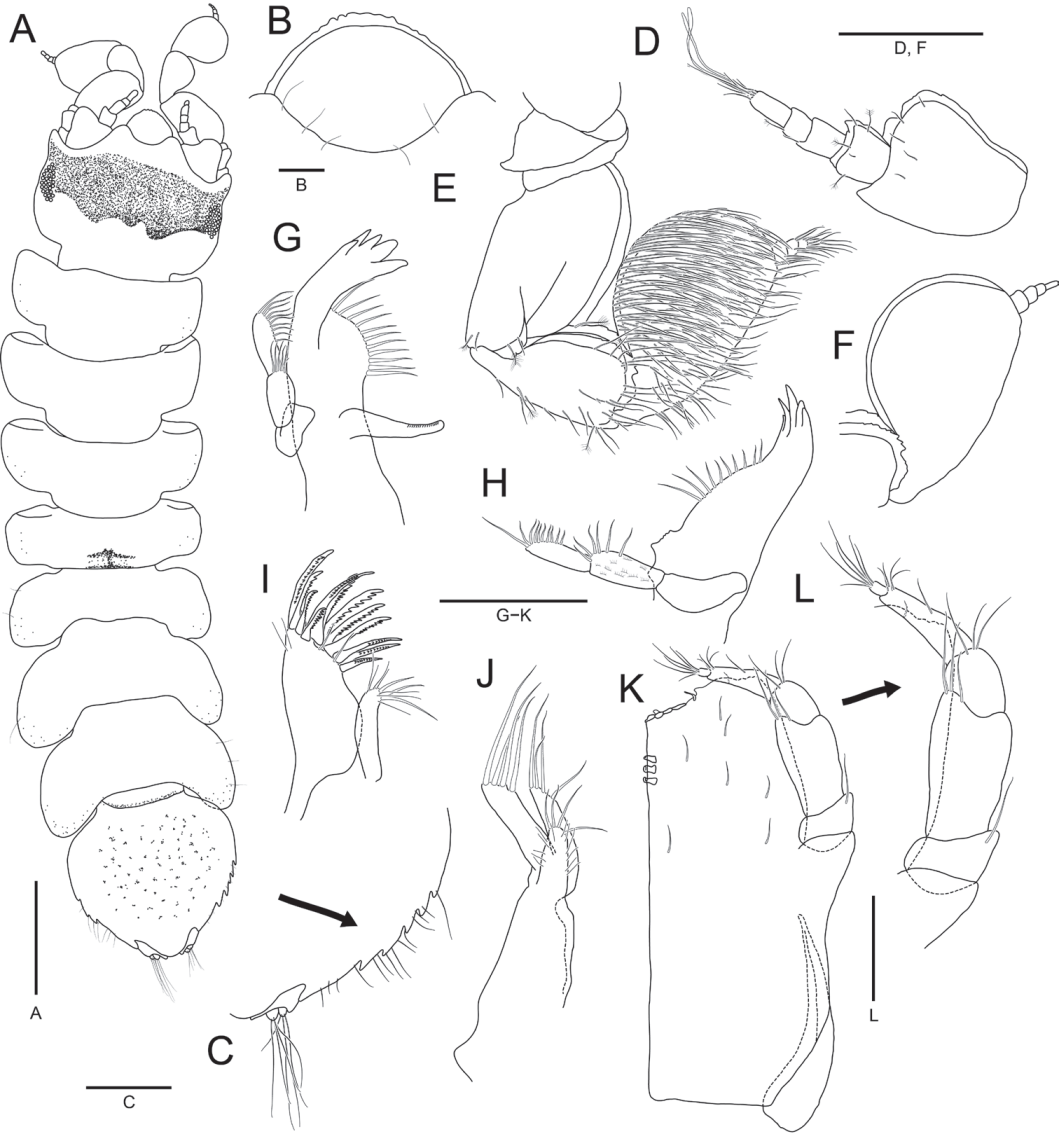
*Joeropsissemicircularis* sp. nov., holotype, male **A** habitus, dorsal view **B** pseudorostrum **C** lateral margin of pleotelson **D** antennula **E** antenna **F** flagellar articles of antenna except for setation **G** left mandible **H** right mandible **I** maxillula **J** maxilla **K** maxilliped **L** detail of maxillipedal palp. Scale bars: 0.5 mm (**A**); 0.2 mm (**C–K**); 0.1 mm (**L**); 0.05 mm (**B**).

***Body*** (Figs 2B, 6A) almost 4.4 × longer than width; dorsal surface matt and smooth, without setae. ***Cephalon*** 0.8 × as long as wide; lateral margins parallel. ***Pseudorostrum*** (Fig. 6B) 0.7 × as long as proximal wide, narrowing anteriorly; apex rounded and rough. Eyes positioned sublaterally, bulging. ***Pereonites*** not compact, widely spaced, with smooth lateral margins. ***Pleotelson*** (Fig. 6C) 1.1 × longer than width, almost globular, tapering on posterior region; lateral margin serrated, with simple setae; caudomedial lobe rounded distally.

***Antennula*** (Fig. 6D), peduncular article 1 square to globular, with cuticular scales along with outer margin; article 2 subsquare, 0.3 × as long as peduncular article 1, with 3 penicillate setae, 2 simple setae, and serrated cuticular scales distally; article 3 0.8 × as long as article 2; flagellar article 1 0.6 × as long as peduncular article 3, with 1 penicillate seta distally; article 2 1.8 × longer than flagellar article 1, with simple setae distally; article 3 minute, with 2 aesthetascs and simple setae on distal end. ***Antenna*** (Fig. 6E, F) composed of 6 peduncular articles and 5 flagellar articles; peduncular article 3 with 1 process on medial margin, article 5 about 1.7 × longer than articles 1–4 combined, with cuticular scales laterally, and 2 simple setae and 2 penicillate setae distally; article 6 0.7 × as long as article 5, with cuticular scales along with distolateral margin; flagellum with numerous simple setae; flagellar article 1 semi-circular, 3.8 × longer than flagellar articles 2–5 combined, 1.3 × longer than peduncular article 6, with cuticular scales on convex margin.

***Mandibles*** (Fig. 6G, H), molar process finely serrate in distal half; spine row with 11 serrate setae in left mandible but 10 in right mandible, and incisor with 5 cusps; palp article 2 with 6 serrate setae distally, article 3 with 10 serrate setae along with lateral margin. ***Maxillula*** (Fig. 6I) inner lobe with 3 robust simple setae and several fine setae distally; outer lobe with 12 strongly serrate robust setae and 2 simple setae distally. ***Maxilla*** (Fig. 6J), inner lobe shorter than 2 outer lobes, with 4 simple setae distally and several fine setae laterally; mesial and outer lobes with 4 serrate setae on distal end, respectively. ***Maxilliped*** (Fig. 6K, L), endite almost 1.1 × longer than greatest width, reaching proximal third of palp article 4, truncated distally; distal margin with 4 tubercular robust and 2 short simple setae medially; medial margin with 3 coupling hooks distally; palp article 2 2.4 × longer than article 1, distomedial margin produced, with 3 simple setae distally; article 3 almost 0.5 × as long as article 2, with 3 setae distally; article 4 1.4 × longer than article 3, 2.8 × as long as wide, tapering distally, with 4 simple setae distally and 1 simple seta laterally; article 5 minute, with 6 simple setae distally; epipod ~ 4.3 × longer than basal width; tapering distally; apex subacute.

**Figure 7.**
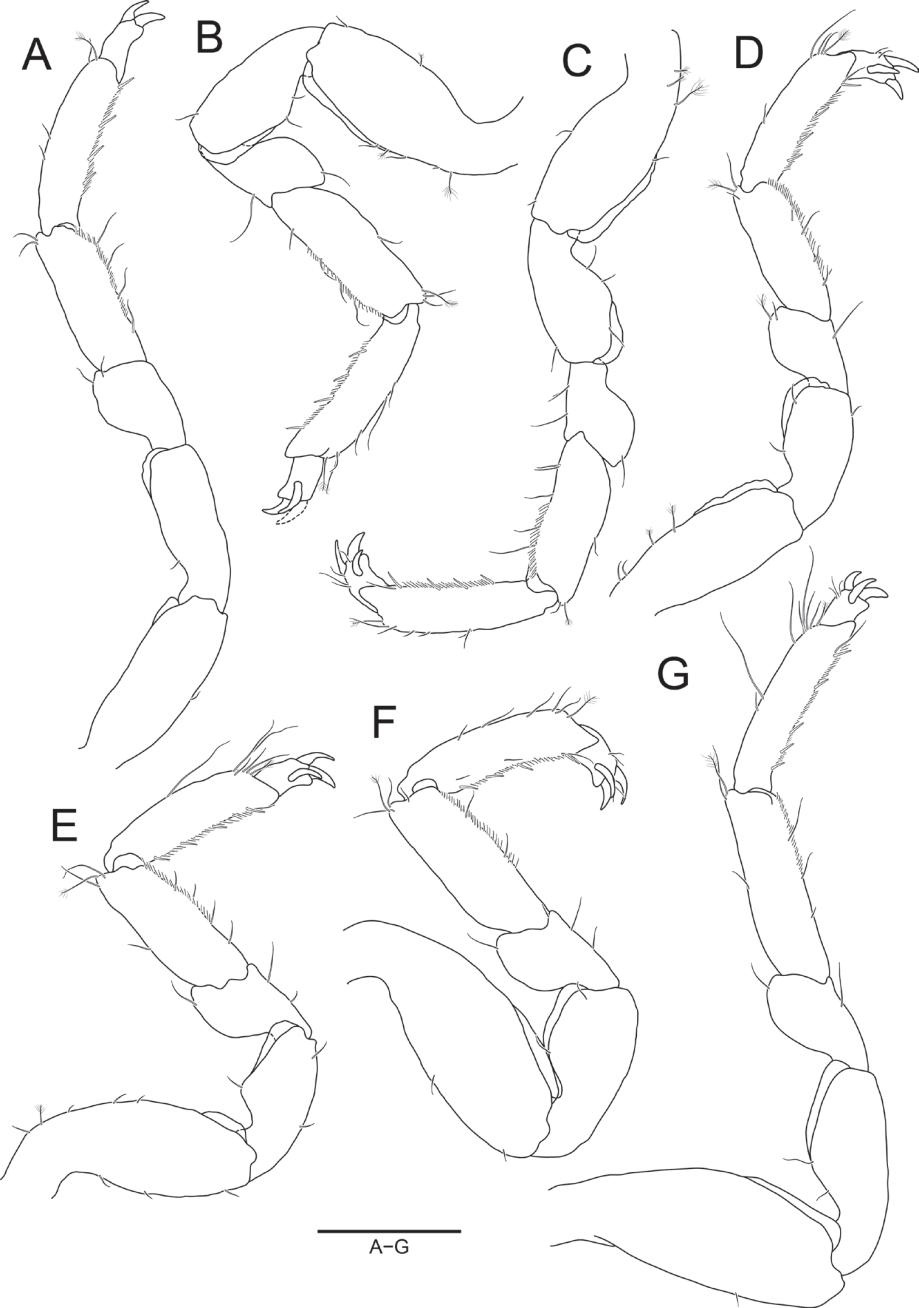
*Joeropsissemicircularis* sp. nov., holotype, male **A** pereopod 1 **B** pereopod 2 **C** pereopod 3 **D** pereopod 4 **E** pereopod 5 **F** pereopod 6 **G** pereopod 7. Scale bar: 0.2 mm.

***Pereopods*** (Fig. 7A–G), basis and ischium with cuticular scales on superodistal end; carpus with cuticular scales inferodistally and numerous short simple setae on inferodistal end; propodus with 2–4 robust setae and numerous short simple setae along with inferior margin. ***Pereopod 1*** (Fig. 7A), basis 2.7 × longer than width, with 1 simple seta on inferior margin; ischium 0.8 × as long as basis; merus 0.6 × as long as ischium, narrowing proximally; carpus 1.6 × longer than merus; propodus 1.2 × longer than carpus, with 1 penicillate seta superiorly; dactylus 0.3 × as long as propodus, with 2 claws on distal end. ***Pereopods 2–7*** (Fig. 7B–G) similar to each other; basis with penicillate setae and simple setae on both lateral margins; ischium ~ 0.8 × as long as basis, convex on superior margin; merus 0.6 × as long as ischium, tapering proximally; carpus subequal to propodus in length, with penicillate setae superodistally; propodus with 1 penicillate seta on superior margin; dactylus with 3 claws and few simple setae distally.

**Figure 8.**
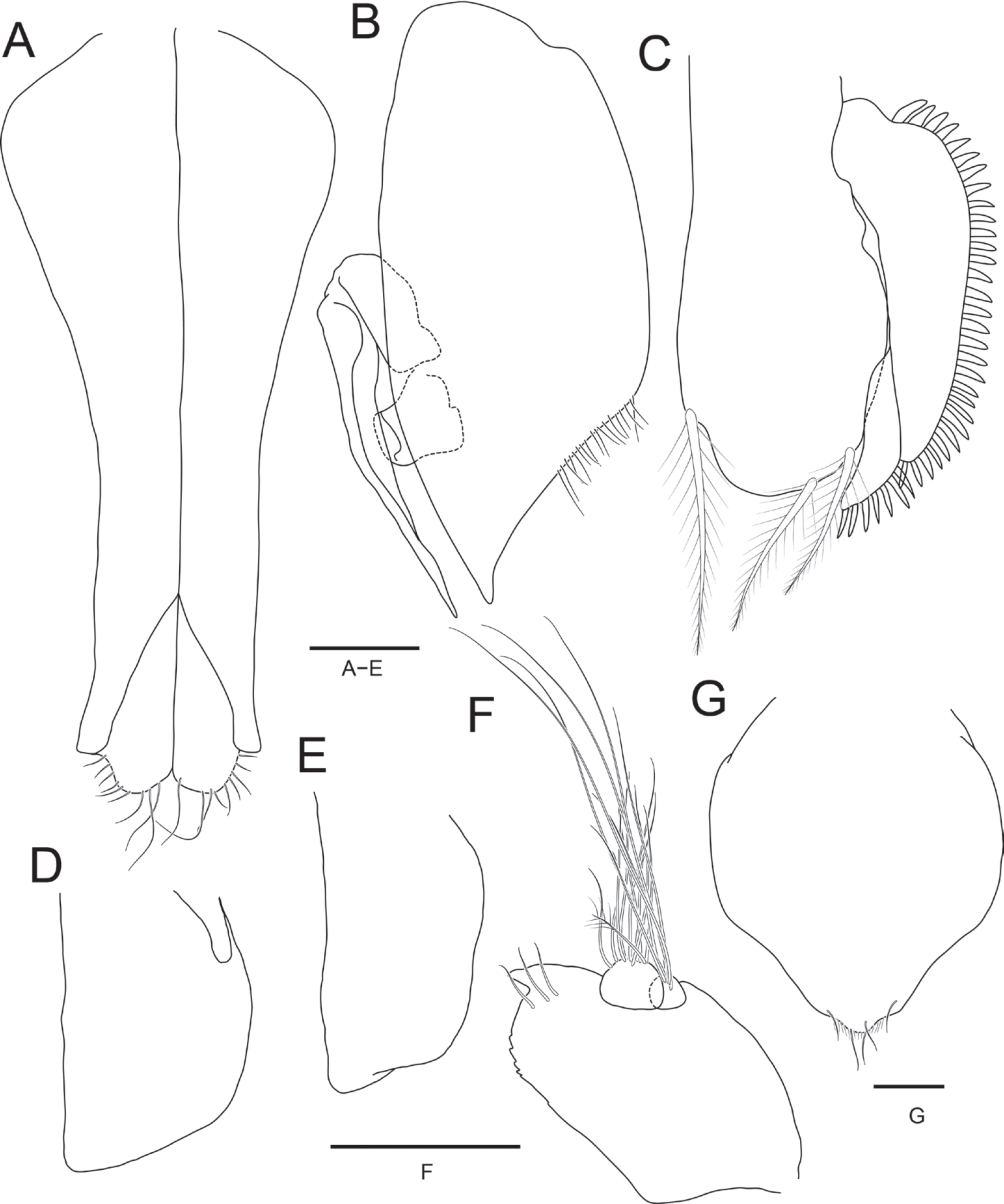
*Joeropsissemicircularis* sp. nov., holotype, male **A** pleopod 1 **B** pleopod 2 **C** pleopod 3 **D** pleopod 4 **E** pleopod 5 **F** uropod. Paratype, female **G** pleopod 2. Scale bars: 0.1 mm.

***Pleopod 1*** (Fig. 8A) 2.3 × longer than greatest width; lateral margins concave; distolateral lobe pointed, not extending distally; apical lobe rounded, with several simple setae. ***Pleopod 2*** (Fig. 8B), protopod 2.3 × longer than greatest width, concave on subapical region, acute distally, with several fine setae on subapical region; endopod positioned at 0.6 length of protopod from proximal region; exopod curved outwardly; appendix masculina acute, extending to apex of protopod. ***Pleopod 3*** (Fig. 8C), endopod 2.1 × longer than half-width, with 3 plumose setae distally; exopod composed of 2 articles, with cuticular scale-setae along with lateral margin, subacute distally. ***Pleopod 4*** (Fig. 8D), endopod 1.5 × longer than greatest width, truncated distally; exopod vestigial. ***Pleopod 5*** (Fig. 8E) without exopod; endopod 1.9 × longer than basal width, truncated distally.

***Uropods*** (Figs 6A, 8F), protopod extending to distal end of pleotelson, medial margin slightly serrated; distomedial end strongly produced and acute, with 3 simple dorsal setae; endopod with 1 penicillate seta and several simple setae distally; exopod smaller than endopod, with several long simple setae distally.

###### Description of female.

Female similar to male. ***Pleopod 2*** (Fig. 8G) 1.2 × longer than greatest width, globular, narrowing proximally, tapering distally; apex broadly rounded, with 4 simple setae apically.

###### Size.

Males 3.7–4.0 mm, mean 3.9 mm (*n* = 4); females 3.3–3.7 mm, mean 3.5 mm (*n* = 6); all from type series.

###### Colour pattern.

The cephalon has a transverse dark brown band of chromatophores across the middle of the cephalon. Pereonite 4 and pleotelson are also covered with chromatophores, but indistinct (Figs 2B, 6A).

###### Variation.

The colour pattern of chromatophores varies according to individual. On the cephalon, a transverse dark brown band is always distinct and regular, while on pereonite 4 or pleotelson, the chromatophores are occasionally varied according to the individuals.

###### Distribution.

South Korea (southern coast of the Korean Peninsula and East Sea).

###### Remarks.

*Joeropsissemicircularis* sp. nov. can be identified by the following features: (1) the body has dark brown chromatophores on the cephalon, pereonite 4, and pleotelson; (2) lateral margins of the cephalon are smooth and parallel; and (3) the first flagellar article of the antenna is swollen and semi-circular in shape.

*Joeropsissemicircularis* sp. nov. resembles four known species by having the cephalon laterally smooth and parallel, the pseudorostrum not concave or pointed distally, maxillipedal palp article 2 lacking medial lobe, and the pleotelson and uropods both laterally serrated: *J.dollfusi* Norman, 1899; *J.latiantennata* Nunomura, 1999; *J.stebbingi* Kensley, 1975; and *J.wolffi* Müller, 1991 (Koehler 1885; Norman 1899; Amar 1961; Kensley 1975; Müller 1991; Nunomura 1999). Among these species, *J.semicircularis* sp. nov. is most similar to *J.latiantennata* by having swollen and semicircular-shaped first flagellar article of the antenna (Nunomura 1999). However, the former differs from the latter by the following characteristic features: (1) the dactylus of pereopods 2–7 has three claws (vs. two claws in the latter); (2) the second peduncular article of the antenna has a process on medial margin (vs. has not in the latter); and (3) the fourth peduncular article of the antenna is not serrated (vs. serrated in the latter) (Nunomura 1999). The new species can be easily distinguishable from the remaining three species by having swollen and semi-circular-shaped first antennal flagellar article (Koehler 1885; Norman 1899; Amar 1961; Kensley 1975; Müller 1991). A detailed comparison of *Joeropsis* species mentioned above is provided in Table 2.

### ﻿Key to known *Joeropsis* species in the Far East

**Table:** 

1	Cephalon with anteriorly narrowing lateral margins	**2**
–	Cephalon with parallel lateral margins	**5**
2	Lateral margins of cephalon serrated	***J.lata***
–	Lateral margins of cephalon not serrated	**3**
3	Antennal peduncular articles 3 and 5 each with a process on medial margin	***J.denticulatus* sp. nov.**
–	Antennal peduncular articles 3 and 5 without any processes	**4**
4	Maxillipedal palp article 2 with medial lobe	***J.affinis***
–	Maxillipedal palp article 2 without medial lobe	***J.lobota***
5	Peduncular article 5 of antenna serrated on outer margin	***J.latiantennata***
–	Peduncular article 5 of antenna not serrated on outer margin	***J.semicircularis* sp. nov.**

## Supplementary Material

XML Treatment for Joeropsis

XML Treatment for Joeropsis denticulatus

XML Treatment for Joeropsis semicircularis
